# Enhancing Physical Literacy Domains Through the Spectrum of Teaching Styles in Recess-Based Active Breaks: A Single-Blind Randomized Controlled Trial

**DOI:** 10.3390/children13050634

**Published:** 2026-05-01

**Authors:** Domenico Monacis, Giacomo Pascali, Dario Colella

**Affiliations:** 1 Department of Education and Sports Sciences, Pegaso University, 80143 Naples, Italy; 2 Department of Biological and Environmental Sciences and Technologies, University of Salento, 73100 Lecce, Italy; giacomo.pascali@unisalento.it (G.P.); dario.colella@unisalento.it (D.C.); 3 Scientific Laboratory on Teaching Methods for Physical Education and Motor Assessment, University of Salento, 73100 Lecce, Italy

**Keywords:** active breaks, health promotion, physical literacy, primary school, selective attention

## Abstract

**Highlights:**

**What are the main findings?**

- ABs during recess seem to have the potential to improve motor skill learning and selective attention in primary-school children compared to traditional recess;
- The experimental intervention highlights a positive effect on the affective domain of PL.

**What are the implications of the main findings?**

- Structured PA-based recess represents a sustainable strategy for Health-Promoting Schools, without reducing time spent on curriculum teaching;
- ABs during recess could be considered an inclusive and flexible pedagogical tool for teachers to promote multiple domains of PL.

**Abstract:**

Background: The integration of active breaks during the school day has been widely demonstrated to be effective in counteracting sedentary behaviors. The present study assessed the efficacy of a structured active breaks (ABs) intervention implemented during recess on multiple domains of Physical Literacy (PL) in primary-school children. Methods: A single-blind randomized controlled trial was conducted with 139 children (aged 9–10 years). Classes were randomized into an Experimental Group (EG, *n* = 66) and a Control Group (CG, *n* = 73). The EG participated in an 8-week intervention (six sessions/week, ~10 min) consisting of coordinative and interdisciplinary motor tasks during recess. Pre- and post-intervention assessments included physical fitness (SLJ, 4 × 10 m SR, 6MWT, MBT), gross motor skills (TGMD-2), selective attention (Bell Test), physical activity levels (PAQ-C), physical self-perception (PSP), and enjoyment (PACES). Results: A mixed-design MANOVA revealed a significant multivariate Time × Group interaction (*p* < 0.001). Univariate analyses showed significant improvements in the EG compared to the CG for explosive strength (*p* < 0.001), agility (*p* < 0.001), Gross Motor Quotient (*p* = 0.003), and selective attention (*p* < 0.001). Furthermore, the EG demonstrated significant increases in physical activity levels, self-perception, and enjoyment (*p* < 0.05). No significant gender interaction was found, indicating equal effectiveness for boys and girls. Conclusions: Transforming recess into a structured opportunity for movement through ABs effectively enhances physical, cognitive, and affective domains. This intervention represents a sustainable strategy for Health-Promoting Schools to foster PL and psychophysical well-being without reducing curricular instruction time.

## 1. Introduction

Socio-economic factors [1], and a lack of recreational facilities [2], parks and free-play areas [3], are among the main factors limiting daily practice of physical activity and the progressive increase in sedentary behaviors [4]. According to international findings, low engagement in daily physical activity and playful activities has a significant negative effect on motor development, including the social, psychological and emotional domains too [5,6,7]. Moreover, the COVID-19 pandemic contributed to a further increase in sedentary habits and the related effects that put the psychophysical well-being of the youngest population at risk [8].

This negative trend highlighted the need to identify new strategies to encourage physical activity in different contexts, especially for children and young adolescents, identifying school as an ideal setting to convey positive messages about healthy lifestyles [9].

In this context, the so-called “Health-Promoting Schools” (HPSs) are defined as dynamic institutions that constantly enhance their ability to act as a proactive learning environment, mobilizing resources and stakeholders in an intersectoral and participatory approach to promoting well-being [10].

Through multidimensional interventions ranging from health education to the creation of safe and inclusive environments, the school integrates policies aimed at respecting individual dignity and enhancing the educational success of each student. This operating model transcends mere teaching, extending the goal of improving health to school staff, families, and the entire local community to generate a systemic and sustainable social impact [10].

In recent decades, multi-component projects in Health-Promoting Schools, through the expansion (e.g., pre/post-school activities, active recess), extension (e.g., by increasing the number of weekly hours of physical education or afternoon sports initiatives) and enhancement of opportunities to be physically active (e.g., increasing the availability of sports equipment and tools, different sports activity courses, identifying spaces and environments to be used simultaneously, etc.) [11], have been shown to be sustainable and effective models aimed at promoting active lifestyles [12,13,14,15].

In this framework, active breaks (ABs) are defined as “short bouts of exercise to reduce or break up prolonged sedentary periods and/or increase overall PA to promote health” [16]. In school setting they represent a methodologically sound strategy for integrating physical activity into the curriculum, promoting interdisciplinarity and the creation of meaningful learning environments [17]. Active breaks are defined as short periods of physical activity during lessons, aimed at interrupting sedentary behavior, improving attention, and promoting a positive classroom atmosphere. Through the proposal of ABs, teachers can help foster motor skill learning and physical fitness, while strengthening transversal skills and disciplinary learning through the reworking of content in dynamic and cooperative contexts [18]. As suggested by recent scientific findings, they are not just “breaks to let off steam,” but they become an integral part of teaching, supporting and reinforcing learning processes, consolidating the role of active breaks (ABs) as an effective strategy in the school setting to promote cognitive functions, physical fitness, motor development and classroom behavior [16,19].

Active breaks have been shown to improve different areas and domains of children’s lifestyles and health (i.e., reducing sedentary behaviors), increasing levels of physical activity at school, and enhancing attention and cognitive functions [20,21,22]. There is also evidence about their effectiveness in relation to cognitive and academic performance [23,24]. Moreover, evidence indicates that implementing short sessions of physical activity in the classroom has a positive impact on on-task behavior and executive functions, suggesting an acute improvement in academic engagement and a reduction in disruptive behaviors [25,26].

A recent systematic review and meta-analysis revealed limited positive acute and chronic effects of active breaks on attentional outcomes, pointing out the key roles of the activity’s intensity and the teacher’s leadership [27].

Although AB-based intervention at school is often associated with an improvement in cognitive function and classroom behavior, their contribution to physical fitness development—as a health indicator [28,29]—should not be underestimated. The increased time spent in moderate-to-vigorous physical activity (e.g., 4–15 min of high-intensity active breaks) contributes to achieving the international recommendation of 60 min of daily MVPA [30], acting as a counterbalance to the prolonged sedentary lifestyle typical of the school environment.

Findings revealed a positive association between time spent in daily physical activity and physical fitness development [31,32,33]. Moreover, while the effectiveness of ABs in increasing energy expenditure and exercise intensity is well documented, evidence regarding their effect in improving physical fitness components (i.e., cardiorespiratory fitness, agility, muscular strength, flexibility, etc.) is still limited [34].

In addition to their metabolic and behavioral impact, active breaks (ABs) represent a strategic opportunity to counteract the decline in motor competence during school age, acting as “windows of practice” for learning fundamental movement skills [17]. While curricular physical education remains the fundamental pillar for structured motor learning, ABs allow for increased exposure to gross motor coordination and fine motor tasks. Recent studies in this field indicate that well-designed AB-based interventions integrating coordinative engaging activities (i.e., jumping, skipping, jumping jacks, and a combination of postural patterns) directly stimulate the neuromuscular circuits responsible for postural control and dynamic balance [35,36]. However, research in this field is still limited and future studies are needed to better understand the role of ABs in soliciting motor development and motor skill learning.

**Figure 1:**
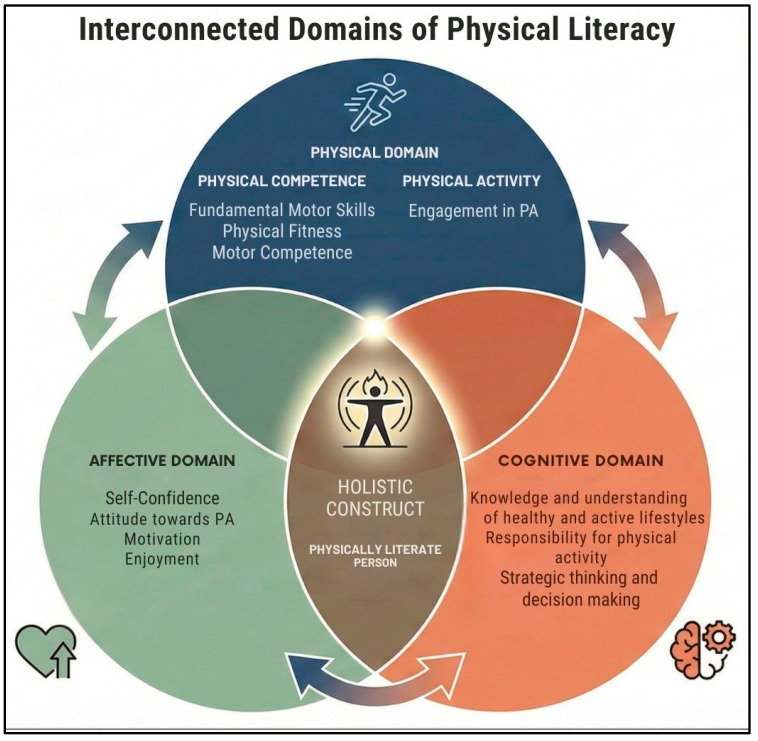
The Multidimensional Framework of Physical Literacy.

Moreover, while cognitive impact and classroom behavior—with their rich literature focusing in particular on selective attention—appear promising, the role of active breaks in structurally improving physical fitness and motor development remains a subject of debate [34,37]. A significant limitation in this area of research is the methodological heterogeneity and poor quality of many studies, which are often characterized by small samples or the absence of blinding in the detectors, factors that can introduce observational bias [27,34,37,38]. To potentially overcome these limitations and ensure a more comprehensive analysis of the potential benefits of AB interventions, this study establishes the Physical Literacy (PL) construct as a scientific and methodological framework for designing programs and interventions aimed at promoting health, preventing disease, and encouraging sport and leisure activities [39]. PL is a holistic construct used worldwide to design high-quality physical education interventions to enhance the child’s motor, cognitive and affective domains [40,41]. It encompasses motivation, self-confidence, motor skills and knowledge, enabling individuals to consciously acquire and maintain physically active lifestyles (Figure 1).

Despite the extensive literature on PL and well-documented benefits of ABs, a significant gap remains in the current literature regarding the pedagogical strategies used for their delivery. A growing body of evidence in physical education suggests that the systematic variation in instructional models, with particular reference to the Spectrum of Teaching Styles (STS), is highly effective in developing multiple dimensions of Physical Literacy. According to this model, reproductive styles are foundational for developing motor competence and physical efficiency, while productive styles are crucial for fostering cognitive engagement, autonomy and intrinsic motivation [42,43,44,45]. However, the translation of this robust pedagogical framework into the specific, short-bout context of active breaks has not been thoroughly investigated. Most studies still predominantly focus on the physiological dosage of the intervention (e.g., duration, intensity and frequency) [22,24,46,47].

To systematically investigate the impact of AB intervention, this study was guided by the following research questions (RQs) and corresponding hypotheses (H), framed within the multidimensional Physical Literacy model.

**RQ1:**  *Does the implementation of an 8-week AB intervention, structured around reproductive teaching styles, significantly improve children’s physical fitness, gross motor skills and daily physical activity behaviors compared to traditional recess?*

**H1:**  *The authors hypothesized that the EG would show significant improvements in fundamental movement skills and fitness components compared to the CG.*

**RQ2:**  *To what extent does the cognitive load required by the productive teaching styles stimulate greater neural engagement, leading to significant enhancements in selective attention?*

**H2:**  *The authors hypothesized that the cognitive load required by the productive teaching styles would stimulate greater neural engagement, leading to significant enhancements in selective attention.*

**RQ3:**  *How does the deliberate variation in teaching styles across the Spectrum influence movement-related psychological factors?*

**H3:**  *The authors hypothesized that the inclusive and varied nature of the teaching styles would lead to significant increases in physical self-perception and enjoyment.*

## 2. Materials and Methods

### 2.1. Participants and Study Design

A single-blind randomized experimental design was used to evaluate the effects of active breaks during recess on motor development and movement-related factors in a sample of primary-school children. Participants (six primary-school classes) were recruited through simple random sampling from a primary school in the Province of Lecce (Italy). The only exclusion criteria were the presence of injuries or any other physical reason that could prevent participation in physical activities. At baseline, 145 students were identified as possible eligible participants. During the experimental procedures, however, participants who missed at least 8 AB sessions (out of a total of 48 sessions) were excluded from the statistical analysis. No participants were excluded from the project for this reason. However, since six of them (2 boys and 4 girls) had certified physical impairments, the final sample was made of 139 students aged 9–10 yrs. After the baseline assessment, three classes were randomly assigned to the Experimental Group (EG = 66) and three to the Control Group (CG = 73) using a random number generator. Participants’ anthropometric characteristics are summarized in Table 1.

A priori power analysis was conducted using G*Power software (version 3.1) [48,49] to determine the minimum sample size required for the study (N = 23, effect size ~ 0.80, α = 0.05 and statistical power = 0.95).

The school principal and teachers received an information statement and were informed of the purpose of the study. Moreover, informed consent was obtained from all parents/legal guardians of children participating in the study. All students participated in the experimental activity as part of a whole-class intervention. This study was approved by the Pegaso University Ethics Committee (PROT/E 002466 of 29 March 2024).

### 2.2. Study Design and Intervention Protocol

**Figure 2:**
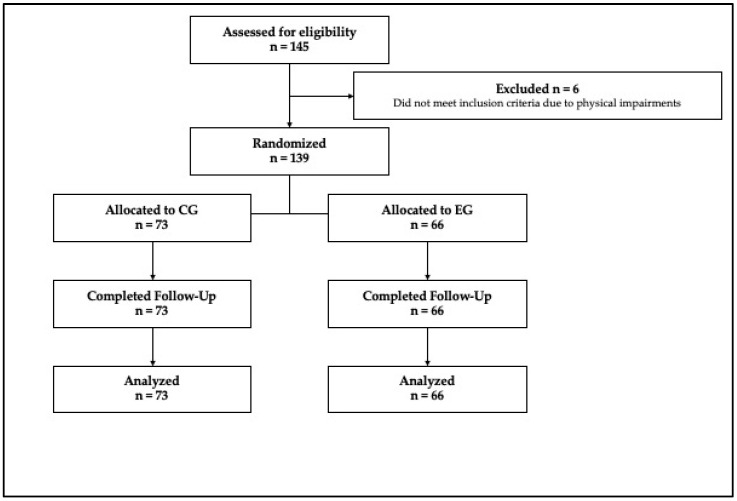
CONSORT flow diagram.

A summary of the study procedure is illustrated in the CONSORT flow chart (Figure 2).

The Control Group (CG) spent recess in the traditional way, while in each class of the Experimental Group (EG), three interventions per week were carried out twice a day for eight weeks in six primary-school 5th-grade classes, for a total of 48 ABs.

According to the existing literature, evidence suggests that 6-to-8 weeks of short bouts of coordinative exercises are adequate to induce early physical and cognitive adaptations [50,51]. Moreover, from a pedagogical point of view, 8 weeks of ABs represent a sustainable and highly realistic model for Health-Promoting Schools [25,26,27], allowing teachers to establish new active routines without experiencing intervention fatigue or compromising the time allocated to curricular subjects.

In the EG, the teacher proposed motor tasks using different tools, including technological ones (dice, spin wheels, interactive whiteboards, LEDs with sensors, etc.), integrating mainly motor tasks with content related to other disciplines.

Experimental interventions were structured around six different thematic areas: (1) rhythm ability; (2) kinesthetic differentiation; (3) motor and postural patterns; (4) motor combination; (5) balance; (6) interdisciplinary active breaks.

A highly innovative pedagogical element of this study is the deliberate application of the Spectrum of Teaching Styles to the design of the ABs. By dynamically shifting from reproductive (e.g., practice, command) to productive teaching styles (e.g., guided discovery, convergent and divergent production), the intervention aimed to move beyond basic physical activation to foster deep motor–cognitive engagement.

ABs were structured to be performed both individually and in pairs, and motor tasks were proposed combining production and reproduction teaching styles [52,53]. To maximize physical, cognitive and affective engagement, each AB session systematically combined two different teaching styles—integrating both reproduction and production styles—by proposing motor tasks in different modalities, as detailed in Table 2.

Moreover, each active break lasted ~10 min, divided into 3 phases:

- Activation ~1–2 min;
- Central phase ~5–6 min;
- Deactivation ~1–2 min.

Four active breaks were developed for all 6 thematic areas and were undertaken on a rotating basis throughout the weeks. The interventions carried out each week and the tools used are listed in Table 2. Experimental activities were performed and supervised by external experts with a Master’s Degree in Preventive and Adapted Sports Sciences at two specific times during the school day: during the first recess (10:00 a.m.) and during the second recess (12:00 p.m.). Before starting experimental activities, the external experts received dedicated theoretical and practical training. It focused on the application of the Spectrum of Teaching Styles and the management of the AB protocol for a total of 20 h (5 days of training × 4 h each) to better ensure the alignment of the intervention according to the designed pedagogical constraints.

To ensure developmental appropriateness, ABs built upon fundamental movement skills already within the children’s prior experience. Novelty and cognitive load were progressively introduced by manipulating environmental constraints (e.g., using reaction lights, multi-tasking, etc.) and varying executive variants. Furthermore, both motor tasks and interdisciplinary contents were strictly co-designed with the classroom teachers to ensure their alignment with specific learning objectives and competence development according to the Italian National Guidelines for Primary School and First Cycle of Education [54].

Intervention fidelity was also assessed through both teachers’ logbooks and direct observations. Teachers were asked to complete weekly logs to verify adherence to the scheduled intervention (48 ABs). Additionally, external trained observers used a specific standardized checklist to assess the pedagogical delivery during a randomly selected 30% of the AB sessions [44]. The overall fidelity score across the observed sessions was 85%, suggesting a high degree of implementation accuracy.

### 2.3. Assessment Procedure and Tools

To investigate the domains of Physical Literacy, different but complementary assessment tools were used. Pre- and post-intervention assessments were conducted one week before the start and one week after the end of the 8-week experimental activities. Both baseline (t_0_) and follow-up (t_1_) assessment were conducted by outcome assessors—specialists holding a Master’s Degree in Preventive and Adapted Sports Activities—who were strictly blinded to the participants’ group allocation to minimize measurement bias.

Furthermore, data entry and basic statistical computations were conducted by researchers who were blinded to the group codes until preliminary analyses were finalized.

Due to the active nature of the intervention, blinding of participants and teachers was not possible. To ensure methodological rigor and data integrity, avoiding potential carryover effect, the evaluation sessions were administered in a systematic order: first, the cognitive domain was assessed, followed by the affective and behavioral domains, and finally, the Physical Domain.

To prevent fatigue and ensure peak performance, physical assessment was administered in the following chronological order: Test of Gross Motor Development-2 (TGMD-2), 1 kg medicine ball throw, standing long jump, 4 × 10 m shuttle run, and the 6-Minute Walking Test. This sequence was chosen to distribute the physical load during assessment.

#### 2.3.1. Physical Domain of Physical Literacy

Physical Competence components of PL were assessed with well-known validated and reliable tests from the literature:

- Standing long jump (SLJ): Assesses explosive strength in the lower body and bilateral coordination [55]. Distance covered by jumping was reported in meters (m).
- The 4 × 10 m shuttle run (SHR): Assesses speed of movement and coordination when changing direction [56]. Time needed to complete the test was reported in seconds (s).
- The 1 kg medicine ball throw (MBT): Assesses upper limb coordination, strength, and object manipulation skills [57]. Distance covered by throwing was reported in meters (m).
- The 6-Minute Walking Test (6WT): Assesses endurance and functional capacity [58]. Distance covered by walking was reported in meters (m).

Children were asked to perform two attempts of SLJ, MBT and 4 × 10 SHR, respectively, while the 6MWT was performed once to prevent fatigue in primary-school children. Only the best result was considered for further analysis.

Gross motor skills were assessed using the Test of Gross Motor Development-2 (TGMD-2), with the appropriate recording sheets for the examiner [59]. The setting required specific equipment, including various types of balls (basketballs, soccer balls, tennis balls, softballs, 4-inch light balls, and 8–10-inch game balls), a beanbag, colored marking tape, traffic cones, and a plastic bag with a batting tee. The protocol measures 12 skills divided into two subtests: 6 locomotion tests (running, galloping, skipping, bouncing, standing long jump, side slipping) and 6 object control tests (striking a stationary ball, stationary dribble, catching, kicking, overhand throw, underhand roll). Performance criteria varied by locomotor (five for hopping, three for jumping in extension, four for the rest) and object control skill (five criteria for batting, three for catching, four for the others).

The administration protocol calls for two trials for each skill. The assessment uses a dichotomous system: a score of 1 is assigned for correct execution of the criterion and 0 for failure.

The sum of the scores for the criteria in the two trials generates the skill score. The sum of the skill scores determines the raw subscale score (range 0–48). Using the normative tables in the TGMD-2 manual, raw scores are converted into standard scores (1–20) and percentiles, stratified by age and gender. The sum of the standard scores of the two subscales is converted into the Gross Motor Quotient (GMQ) (range 46–160). Finally, the level of motor competence is categorized using seven qualitative descriptors, ranging from “very poor” to “very superior,” applicable to both the standard scores of the subscales and the overall GMQ. In this study, only the GMQ was used by researchers as a measure of total score, combining the standard scores from the locomotor and object control subtests. Despite the observational nature of the assessment, the use of strict standardized performance criteria evaluated by blinded specialists significantly mitigated the risk of subjective observation bias, ensuring high data validity.

Moreover, according to the holistic framework of Physical Literacy, engagement in physical activity represents one of the key components of Physical Domain.

Daily physical activity was used to assess behavioral manifestation of Physical Domain using the Italian version of the PAQ-C questionnaire [60,61,62]. The questionnaire consists of 9 multiple-choice questions aimed at providing a general estimate of the level of moderate and vigorous physical activity performed during the 7 days prior to completing the questionnaire. It does not measure physical activity in quantitative terms (minutes or METs), but returns a physical activity index on a scale of 1 to 5, where 1 indicates a very low level and 5 a very high level.

#### 2.3.2. Affective Domain of Physical Literacy

The movement-related factors and cognitive functions were assessed as follows:

- Physical self-perception (PSP) [63]: The questionnaire consists of six items that assess aspects such as speed, strength, and coordination as perceived by the children. Each item presents a statement relating to a specific physical skill, and the child is asked to indicate the degree to which they feel competent in that particular skill. Responses are generally provided on a 4-point Likert scale, where higher scores indicate greater perceived self-efficacy. The sum of the 6 items generates the overall score.
- Enjoyment (PACES) questionnaire [64]: Designed to assess the degree of pleasure or enjoyment an individual experiences during physical activity. The Italian adaptation consists of 16 items, 9 of which are formulated in a positive way and 7 in a negative way. Each item is evaluated on a 5-point Likert scale, where higher scores on positive items (PACES_P) and lower scores on negative items indicate greater enjoyment of physical activities (PACES_N).

#### 2.3.3. Cognitive Domain of Physical Literacy

Selective attention, through the modified Bell Test [65]: This is a paper-and-pencil neuropsychological test used to assess selective and sustained visual attention. The modified version was designed to make it more suitable for certain populations (e.g., developmental age or clinical populations) and to improve standardization. The subject is presented with 4 sheets containing a dense grid of visual stimuli, including: (a) targets: stylized bells; (b) distractors: other similar objects (hourglasses, mushrooms, anchors, etc.). The task is to circle all the bells on the sheet for a total time of 2 min. Assessment: Quick (bells found in the first 30 s, circled with a red pen); total (bells found in 2 min, with a pen of a different color, added to those found in the first 30 s).

### 2.4. Statistical Analysis

Before starting analysis, data were checked to identify possible outliers and test for normal distribution. Descriptive statistics have been reported according to group (CG and EG) and gender (boys and girls) in terms of mean ± standard deviation (M ± SD).

A mixed-design multivariate analysis of variance (MANOVA) was performed to assess the effectiveness of the AB intervention protocol on physical fitness components, motor skills, cognitive functions, and movement-related variables. This analysis design included a 2 (Time) × 2 (Group) MANOVA. Moreover, to assess whether the intervention protocol was moderated by gender, a secondary 2 (Group) × 2 (Time) × 2 (Gender) MANOVA was performed. In these analyses, Time was treated as a within-subjects factor, while Group and Gender were treated as between-subjects factors. Pillai’s Trace was used as the main statistic, as it is more robust in the presence of violations of the assumption of homogeneity of covariance matrices (Box’s M). Following this, mixed ANOVAs for repeated measures were conducted for each variable to examine in detail the effect of the intervention on each dependent variable. Effect sizes were expressed as partial eta-squared (ηp2): ηp2 ~ 0.01 as a measure of small effect; ηp2 = 0.06 as medium effect; and ηp2 ~ 0.14 as large effect size [66]. We calculated 95% Confidence Intervals (95% CIs) for all group differences. Furthermore, the Holm–Bonferroni sequential correction method was applied to all resulting *p*-values.

Significant index was set at *p* < 0.05 for all statistical analysis. All statistical analyses were performed using IBM SPSS Statistics, version 29 (IBM, Chicago, IL, USA).

## 3. Results

Table 3 reports descriptive statistics for both the CG and EG pre- and post-intervention, respectively. Baseline analysis (t0) revealed no significant differences between the CG and EG.

The results of the mixed-design multivariate analysis of variance (MANOVA) are shown in Table 4. The analysis (Pillai’s Trace) revealed that the multivariate interaction Time × Group was highly significant with a very large effect size [F(1.137) = 8.159, *p* < 0.001, ηp2 = 0.48], indicating an overall effect of treatment on the set of dependent variables.

**Figure 3:**
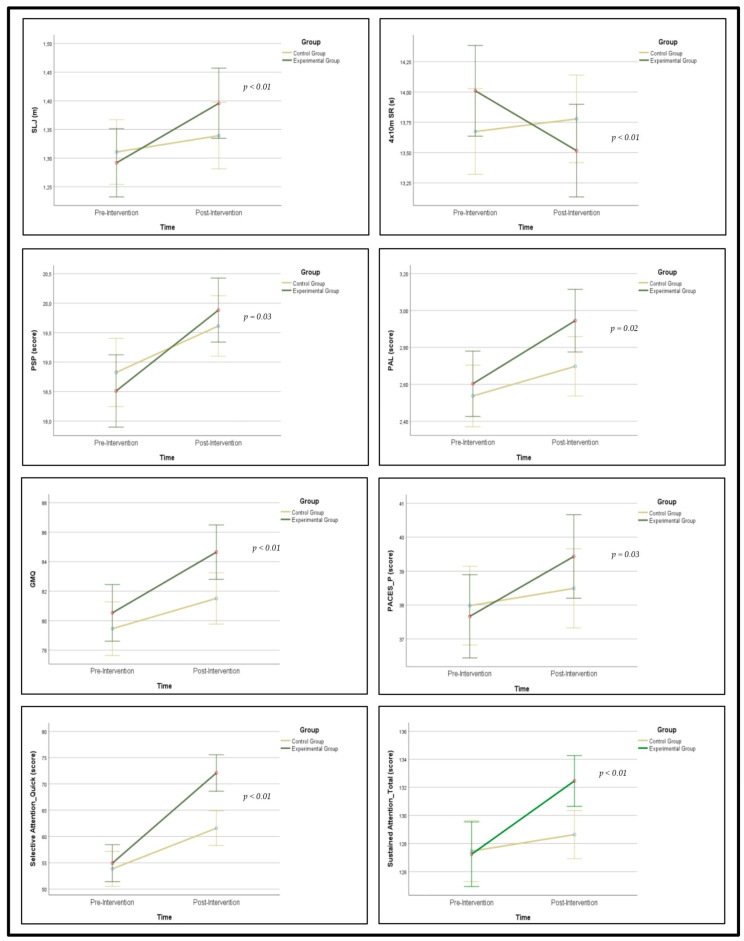
Visual summary of Time × Group interactions.

Subsequent univariate analyses showed significant interactions in favor of the EG for physical fitness and motor skills [SLJ (*p* < 0.001; ηp2 = 0.129), 4 × 10 m SR (*p* < 0.001; ηp2 = 0.172), GMQ (*p* < 0.003; ηp2 = 0.082)], cognitive function [quick (*p* < 0.001; ηp2 = 0.244) and total selective attention (*p* < 0.001; ηp2 = 0.168)], and movement-related variables [PACES_P (*p* = 0.029; ηp2 = 0.04), PAL (*p* = 0.022; ηp2 = 0.049) and PSP (*p* = 0.031; ηp2 = 0.043)]. Graphical representations are shown in Figure 3.

Table 5 summarizes the results of the multivariate analysis conducted to assess the moderating effects of gender on changes in dependent variables across groups. Although a significant main effect of the Gender factor was highlighted [F(11.135) = 3.64, *p* < 0.001, ηp2 = 0.299], suggesting baseline differences between boys and girls, no significant interactions were found for Time × Gender (*p* = 0.573) or for the three-way interaction Time × Group × Gender (*p* = 0.128).

## 4. Discussion

The present study examines the potential role of the AB intervention protocol based on the STS during recess in primary-school children (9–10 years old) for the promotion of Physical Literacy.

Obtained data confirmed our hypothesis, according to which the effectiveness of ABs in the school setting is strictly driven by the pedagogical methodology applied.

The improvements observed in selective attention and the reduction in negative enjoyment confirmed the potential of ABs to positively affect classroom behavior and enjoyment during physical activities, as reported in other similar studies [20,67]. Moreover, when structuring the experimental activities, the combination of duration (~10 min) and frequency (twice a day, three days a week) parameters seemed to have encouraged an optimal state of activation, improving the ability to maintain attention and respond quickly to stimuli. From a pedagogical perspective, these results can be linked to the implementation of productive teaching styles. By requiring children to creatively explore motor solutions rather than merely execute movement instructions, the cognitive load of the intervention was significantly increased, stimulating the executive functions needed for academic attention [21].

The obtained results support the idea that short sessions of physical activity can generate significant cognitive benefits, affecting processing speed and, according to other international findings, academic skills [68,69,70,71,72].

However, one the main results of the study concerns the improvement, in the EG, in Physical Domain (muscular strength, speed and agility, Gross Motor Quotient), suggesting an effectiveness of this kind of intervention to improve skills-related fitness and motor skill learning during childhood.

Given the relatively short duration of the intervention, the significant improvement in explosive strength and agility can be largely attributed to rapid neural adaptations rather than morphological changes. In this sense, the use of reproductive teaching styles during highly coordinative tasks could have enhanced inter- and intra-muscular coordination, leading to measurable gains in functional performance even within a short timeframe.

Despite the observed improvements in other components of physical fitness, cardiorespiratory fitness did not show significant differences between the Experimental and Control Groups over time. This finding is consistent with the specific nature of the experimental protocol adopted: the active breaks were ~10 min in duration and occurred three times weekly, with a primary focus on tasks involving coordination, rhythm, motor combination and postural control. From a physiological perspective, inducing stable adaptations in cardiorespiratory fitness typically requires more sustained volumes of aerobic activity and consistently high intensities, which differ from the intermittent and neuromotor stimuli typical of the “movement snacks” used in this study [73]. These findings suggest that, while ABs represent an effective tool as a “window of practice” for the development of some components of physical fitness (particularly skills-related), gross motor skills and cognitive function, they should not be viewed as a substitute for vigorous physical activity or curricular physical education. Rather, they should be considered a complementary and sustainable strategy for promoting PL in a multidimensional way, without claiming to drastically impact aerobic fitness in such a short time.

There were also positive results concerning the improvement in positive enjoyment during PA, daily levels of physical activity and physical self-perception. These results are consistent with the hypothesis that integrating short periods of movement into the school day can be effective in promoting not only daily levels of physical activity, but also psycho-cognitive and motivational domains [74,75].

The findings regarding the improvements in physical fitness is the subject of an international debate: some authors have highlighted the effectiveness of ABs in enhancing physical fitness components in school children and/or adolescents [35,46,74,75], while others reported non-significant fitness improvements [47].

From a Physical Domain point of view, the improvements observed in motor skill learning are consistent with other findings in the literature [35,36,73,74,75,76,77], highlighting the feasibility of active break intervention in producing significant increases in overall Physical Literacy domains and motor competence (i.e., jumping, running, and object control skills), even in inclusive settings. Moreover, although our population did not include students with disabilities, the short, frequent, and structured nature of the activities seems to have produced similar benefits in the development of basic motor skills.

The increase in physical self-perception can be attributed to the playful and non-evaluative nature of the intervention, which created a positive and favorable environment. Similar results were observed by Mendoza-Muñoz et al. (2022), who found an increase in motivation towards regular physical activity probably favored the inclusive and cooperative context of the breaks [36].

Regarding the effect of Gender on changes in dependent variables after the intervention across groups, the lack of significance suggests that the active break protocol produced comparable benefits in both sexes, confirming its validity regardless of gender.

Given these conflicting scientific results, the design and development of methodologically sound interventions becomes particularly important. The diversity of the literature indicates that the effectiveness of ABs depends heavily on specific parameters such as the duration of the intervention, frequency, methodology used, the type of tasks offered (e.g., cognitive demands vs physical demands), and strict modulation. Therefore, careful structuring of these variables is necessary not only to maximize the potential benefits to students’ psychophysical health, but also to standardize protocols and clarify the discrepancies currently present in international debates.

The effect size observed holds significant practical and pedagogical relevance. The magnitude of the moderate-to-large effect size found in this study suggests the shift from productive to reproductive teaching styles was not only theoretically sound, but generated a relevant impact on children’s ability to maintain classroom focus and efficiently perform daily motor tasks.

### 4.1. Study Limitations

Some critical issues need to be discussed. The limited sample size (139 students from a provincial context), the short duration of the intervention, and the absence of a medium- to-long-term follow-up could affect the generalizability and validity of the results.

Another critical element for the transferability of such interventions concerns teachers’ perceptions: despite the potential benefits of ABs, there is often a lack of awareness or perceptual barriers regarding the real effectiveness of active breaks, which are often seen as conflicting with curricular requirements, compromising their systematic adoption [78]. According to Porter et al. (2024), one of the main reasons for this ineffectiveness is the lack of attention to the school context in the design, implementation, and evaluation of interventions [38]. The term ‘school context’ refers to both the factors that influence the school as an intervention environment (cultural, social, economic, environmental) and those that affect the individuals who deliver or receive the intervention (demographic, socio-economic). As the context varies considerably from one school to another, it can influence the success of an intervention.

A further significant limitation of this study is related to the class-level randomization. In this study, participants were recruited within classes, so individual observations were not strictly independent. To assess the magnitude of this structure, the interclass correlation coefficient (ICC) was calculated for primary outcomes at baseline. Since the ICC was less than 0.05, it indicates a minimal clustering effect. Future large-scale interventions with a higher number of participating classes should employ mixed-effects modeling to rigorously account for cluster randomization (e.g., via Multilevel Modeling).

Furthermore, increasing the number of clusters will be essential to specifically analyze and isolate the effects of intervention based exclusively on production versus reproduction teaching styles, thereby guaranteeing greater reliability and depth to the findings.

Additionally, the current study design compared the experimental pedagogical intervention against a traditional, unstructured recess. The absence of a third and active Control Group (one performing standard active breaks without the manipulation of teaching styles) prevents us from isolating the added value of the pedagogical framework from the general benefits of ABs. Future research should implement a three-arm randomized controlled trial to improve the results’ reliability.

Finally, although this study implemented rigorous protocols to ensure intervention fidelity—such as standardized teacher training to deliver specific teaching styles—it did not control for several potential confounding variables. Factors such as participants’ socio-economic status, nutritional habits, and fluctuation in extra-curricular sports participation were not tracked during the intervention period. To avoid excessive participant burden and maximize compliance with the ecological school setting, we limited our assessment to the primary PL domains. However, the absence of these lifestyle covariates limits the possibility to perfectly isolate the intervention’s effect, as the unmeasured variables might also influence physical and cognitive development. Future research should consider adopting multidisciplinary assessment to control for these socio-demographic and behavioral confounders.

### 4.2. Research Contributions and Methodological Implications

Teachers play a crucial role in promoting health and active lifestyles in a school setting. However, some challenges need to be discussed: some teachers see ABs as a distraction that takes time away from teaching, while others say they do not have enough space, resources, or time to plan them. To overcome these barriers, training must go beyond simply proposing physical activities and provide methodological and didactic tools that allow breaks to be integrated in a way that is consistent with curricular objectives. This implies the development of skills and competence that allow teachers to adapt motor tasks to different contexts/children. Concrete support is provided by the development of a personal portfolio of motor tasks, which offers teachers a flexible and interdisciplinary repertoire of activities, transforming active breaks into real teaching tools. As suggested by Porter et al. (2024), a new approach called the “flexible portfolio of school interventions” requires each school to select the most appropriate intervention components from a framework in order to build their own customized program [38]. Each school will then be able to create its own ‘portfolio’ by combining components based on local needs, available facilities, priorities, and school culture [38]. Moreover, worldwide experiences in this field (i.e., Switzerland, the United States, Norway, Australia, etc.) confirm that the dissemination and sustainability of these practices can be ensured only through continuous teacher training.

Three main contributions of this study can be highlighted:

- Recess’s educational meaning: unlike most studies that implement active breaks during lessons, our intervention took place during recess, demonstrating that even non-teaching moments can be valued as educational spaces, confirming the operational flexibility of this tool to enhance different domains of PL;
- Interdisciplinarity and non-linear teaching: the design integrated motor and cognitive content through playful and varied methods, in line with the most effective approaches for stimulating attention, motivation, and learning;
- Inclusiveness and sustainability: the results confirm that active breaks are an accessible strategy that can be adapted to different school contexts and are potentially useful for children with special educational needs.

Moreover, interventions based on active breaks must be developed differently depending on the target groups: in a recent study by Yin et al. (2025) it was highlighted that high-intensity protocols are recommended for moderately active young people (e.g., university students or workers), while functional or resistance training is recommended for older adults or patients with chronic and noncommunicable diseases [16]. This evidence reflects the need to adapt the parameters of duration, intensity, frequency, and the type of intervention to the target population, highlighting the heterogeneity of the operational proposals.

## 5. Conclusions

While the present study provides promising evidence that an 8-week high-frequency AB intervention can positively impact the physical, cognitive and affective domains of PL, these findings must be interpreted with caution. The small sample size drawn from a single regional context, the short-term nature of the intervention, and the absence of a longitudinal follow-up cannot lead to generalized results. However, the results strongly suggest a relevant pedagogical insight: the how (systematic application of the Spectrum of Teaching Styles) should not be separate from the physiological how much (dosage). Shifting from reproductive to productive teaching styles offers a valid framework to solicit specific motor and cognitive adaptations during school recess.

Future research should include larger samples, long-time follow-up, and a comparison between different forms of implementation (i.e., during curricular lessons, at recess, in outdoor spaces) and methodologies, in order to further investigate the validity of this pedagogical approach to school-based physical activity.

## Figures and Tables

**Table 1:** Participants’ descriptive profiles.

Group	Gender	N	Age	Weight	Height	BMI
CG	Female	36	9.36 ± 0.59	35.32 ± 7.30	1.39 ± 0.09	18.22 ± 2.69
	Male	37	9.19 ± 0.62	36.34 ± 8.67	1.38 ± 0.07	18.97 ± 3.45
EG	Female	36	9.39 ± 0.79	31.47 ± 7.06	1.35 ± 0.07	17.11 ± 3.18
	Male	30	9.23 ± 0.50	34.38 ± 8.30	1.39 ± 0.07	17.57 ± 3.11
Total		139				

EG: Experimental Group; CG: Control Group.

**Table 2:** Intervention protocol and weekly organization of ABs.

ABs’ Topic	Materials and Methods	Teaching Styles	Weekly Organization
Rhythm (perform executive variants, e.g., alternating–successive–simultaneous, before–after, etc.)	Modified version of the hopscotch game, hand clapping, audio track on the IWB	Command + Practice	Weeks: 1st, 3rd, 5th, 7th
Motor competence: kinesthetic differentiation (perform executive variants, e.g., fast–slow–heavy–light–before–after, etc.)	Reaction lights, hopscotch, dice, rock–paper–scissors	Guided Discovery + Command	Weeks: 1st, 3rd, 5th, 7th
Motor and postural patterns	Reaction lights, dice, video on the IWB	Convergent Production + Practice	Weeks: 2nd, 4th, 6th, 8th
Motor combination	Reaction lights, adapted version of the hopscotch game, dice, rock–paper–scissors game	Convergent + Divergent production	Weeks: 2nd, 4th, 6th, 8th
Balance	Reaction lights, adapted version of the hopscotch game, video on the IWB of the “bridge”, dice, rock–paper–scissors game	Inclusion + Divergent Production	Weeks: 1st, 3rd, 5th, 7th
Interdisciplinary	Video on IWB	Guided Discovery + Inclusion	Weeks: 2nd Geography; 4th History; 6th Italian; 8th English

**Table 3:** Descriptive statistics according to Time and Group.

Variable	Control Group (*n* = 73)		Experimental Group (*n* = 66)		Baseline *p* Value
	t_0_	t_1_	t_0_	t_1_	
Physical Domain	M ± SD [CI 95%]	M ± SD [CI 95%]	M ± SD [CI 95%]	M ± SD [CI 95%]	
SLJ (m)	1.31 ± 0.22 [1.25–1.35]	1.34 ± 0.23 [1.28–1.38]	1.29 ± 0.20 [1.20–1.30]	1.40 ± 0.21 [1.30–1.41]	0.515
4 × 10 m SR (s)	13.67 ± 1.27 [13.46–14.02]	13.78 ± 1.37 [13.52–14.16]	14.01 ± 1.43 [13.82–14.58]	13.52 ± 1.38 [13.35–14.01]	0.663
6MWT (m)	646.18 ± 52.54 [628.95–656.22]	684.11 ± 67.50 [661.56–694.70]	591.86 ± 51.33 [571.50–601.17]	629.10 ± 56.60 [608.50–637.77]	0.871
MBT (m)	3.81 ± 0.86 [3.59–4.01]	4.03 ± 0.88 [3.79–4.23]	3.81 ± 0.90 [3.46–3.90]	4.15 ± 0.84 [3.82–4.23]	0.438
GMQ (score)	79.46 ± 7.50 [76.85–80.46]	81.51 ± 6.60 [79.17–82.30]	80.53 ± 6.20 [77.56–80.33]	84.65 ± 6.68 [82.03–85.35]	0.196
PAL (score)	2.54 ± 0.63 [2.37–2.67]	2.70 ± 0.58 [2.54–2.81]	2.60 ± 0.65 [2.40–2.73]	2.95 ± 0.64 [2.78–3.11]	0.882
Affective Domain					
PACES_P (score)	37.98 ± 4.67 [36.46–38.60]	38.49 ± 4.23 [37.24–39.15]	37.67 ± 4.13 [36.10–38.27]	39.43 ± 4.65 [36.71–40.05]	0.726
PACES_N (score)	10.60 ± 4.11 [9.74–11.63]	10.18 ± 3.41 [9.50–11.06]	9.65 ± 2.86 [9.10–10.50]	9.06 ± 2.82 [8.15–9.59]	0.054
PSP (score)	18.82 ± 2.29 [18.22–19.23]	19.61 ± 1.89 [19.01–19.87]	18.51 ± 2.11 [17.53–18.75]	19.88 ± 2.03 [18.63–20.20]	0.643
Cognitive Domain					
Selective Attention_Quick (score)	53.86 ± 12.99 [51.72–57.86]	61.58 ± 11.01 [59.89–65.17]	54.94 ± 12.25 [50.90–57.43]	72.08 ± 14.08 [65.99–73.54]	0.558
Sustained Attention_Total (score)	127.46 ± 7.50 [126.01–129.36]	128.63 ± 6.63 [127.40–130.38]	127.24 ± 9.07 [123.64–128.94]	132.45 ± 6.42 [129.54–133.40]	0.111

**Table 4:** Multivariate and univariate effects (Time × Group).

Measure	Time × Group		
	F	*p*	ηp2
Multivariate Test (Pillai’s Trace)	8.159	<0.001	0.483
Univariate Tests			
Physical Domain			
SLJ (m)	15.67	<0.001	0.129
4 × 10 m SR (s)	21.98	<0.001	0.172
6MWT (m)	0.01	0.946	0.004
MBT	3.30	0.072	0.03
GMQ	9.42	0.003	0.082
PAL (score)	5.44	0.022	0.049
Affective Domain			
PACES_P (score)	4.93	0.029	0.044
PACES_N (score)	0.13	0.721	0.001
PAL (score)	5.44	0.022	0.049
PSP (score)	4.77	0.031	0.043
Cognitive Domain			
Selective Attention_Quick (score)	34.13	<0.001	0.244
Sustained Attention_Total (score)	21.40	<0.001	0.168

**Table 5:** Summary of gender analysis. F = F-statistic; df = degrees of freedom; *p* = *p*-value; ηp2 = partial eta squared.

Multivariate Effects	Pillai’s Trace	F	df	*p*	ηp2	Interpretation
Gender	0.299	3.64	1.137	<0.001	0.299	Significant baseline differences between boys and girls
Time × Gender	0.092	0.87	1.137	0.573	0.092	Boys and girls improved at similar rates overall
Time × Group × Gender	0.153	155	1.135	0.128	0.153	The intervention was equally effective for both genders

## Data Availability

The data presented in this study are available on request from the corresponding author due to privacy reasons.

## References

[1] Ziegeldorf A., Schoene D., Fatum A., Brauer K., Wulff H. (2024). Associations of Family Socioeconomic Indicators and Physical Activity of Primary School-Aged Children: A Systematic Review. BMC Public Health.

[2] Guerra J., Jhon J., Lanza K., Castro G., Barengo N.C. (2024). The Availability between Recreational Facilities and Physical Activity of US Adolescents. Prev. Med. Rep..

[3] Schipperijn J., Madsen C.D., Toftager M., Johansen D.N., Lousen I., Amholt T.T., Pawlowski C.S. (2024). The Role of Playgrounds in Promoting Children’s Health—A Scoping Review. Int. J. Behav. Nutr. Phys. Act..

[4] Ling F.C.M., Khudair M., Ng K., Tempest G.D., Peric R., Bartoš F., Maier M., Brandes M., Carlin A., Ciaccioni S. (2024). DE-PASS Best Evidence Statement (BESt): Determinants of Self-Report Physical Activity and Sedentary Behaviours in Children in Settings: A Systematic Review and Meta-Analyses. PLoS ONE.

[5] Zhao G., Xiao L., Chen Y., Zhang M., Peng K., Wu H. (2025). Association between Physical Activity and Mental Health Problems among Children and Adolescents: A Moderated Mediation Model of Emotion Regulation and Gender. J. Affect. Disord..

[6] Rodriguez-Ayllon M., Cadenas-Sánchez C., Estévez-López F., Muñoz N.E., Mora-Gonzalez J., Migueles J.H., Molina-García P., Henriksson H., Mena-Molina A., Martínez-Vizcaíno V. (2019). Role of Physical Activity and Sedentary Behavior in the Mental Health of Preschoolers, Children and Adolescents: A Systematic Review and Meta-Analysis. Sports Med..

[7] Burton A.M., Cowburn I., Thompson F., Eisenmann J.C., Nicholson B., Till K. (2023). Associations Between Motor Competence and Physical Activity, Physical Fitness and Psychosocial Characteristics in Adolescents: A Systematic Review and Meta-Analysis. Sports Med..

[8] Zaccagni L., Gualdi-Russo E. (2025). Reduced Physical Activity and Increased Weight Status in Children and Adolescents During the COVID-19 Pandemic: A Systematic Review. Children.

[9] Mazur A., Caroli M., Radziewicz-Winnicki I., Nowicka P., Weghuber D., Neubauer D., Dembiński Ł., Crawley F.P., White M., Hadjipanayis A. (2018). Reviewing and Addressing the Link between Mass Media and the Increase in Obesity among European Children: The European Academy of Paediatrics (EAP) and The European Childhood Obesity Group (ECOG) Consensus Statement. Acta Paediatr..

[10] World Health Organization (WHO) Health Promoting Schools. Overview. https://www.who.int/health-topics/health-promoting-schools#tab=tab_1.

[11] Beets M.W., Okely A., Weaver R.G., Webster C., Lubans D., Brusseau T., Carson R., Cliff D.P. (2016). The Theory of Expanded, Extended, and Enhanced Opportunities for Youth Physical Activity Promotion. Int. J. Behav. Nutr. Phys. Act..

[12] Milton K., Cavill N., Chalkley A., Foster C., Gomersall S., Hagstromer M., Kelly P., Kolbe-Alexander T., Mair J., McLaughlin M. (2021). Eight Investments That Work for Physical Activity. J. Phys. Act. Health.

[13] Ilić A., Rumbak I., Brečić R., Colić Barić I., Bituh M. (2023). Three-Year School-Based Multicomponent Intervention May Change Fruit and Vegetable Preferences in Primary School Children-A Quasi-Randomized Trial. Nutrients.

[14] da Costa R.M., Lopes M.V.V., da Costa B.G.G., Malheiros L.E.A., dos Santos P.C., Arundell L., da Silva K.S. (2025). Effect of a School-Based Multicomponent Intervention on Time-Segmented Physical Activity and Sedentary Behavior among Adolescents: A Cluster Randomized Control Trial. BMC Public Health.

[15] Bandera-Campos F.J., Grao-Cruces A., Camiletti-Moirón D., Martín-Acosta F., Muñoz-González R., González-Pérez M., Ruiz-Hermosa A., Vaquero-Solís M., Padilla-Moledo C., Sánchez-Oliva D. (2025). Effectiveness of a Multicomponent Intervention to Promote Physical Activity during the School Day: Rationale and Methods of the MOVESCHOOL Study. Front. Public Health.

[16] Yin M., Li Y., Aziz A.R., Buffey A., Bishop D.J., Bao D., Nassis G.P., Islam H., Wang H., Fyfe J.J. (2025). Short Bouts of Accumulated Exercise: Review and Consensus Statement on Definition, Efficacy, Feasibility, Practical Applications, and Future Directions. J. Sport Health Sci..

[17] Lander N.J., Contardo Ayala A.M., Mazzoli E., Lai S.K., Orr J., Salmon J. (2024). Beyond “Brain Breaks”: A New Model for Integrating Classroom-Based Active Breaks. J. Phys. Educ. Recreat. Danc..

[18] Bailey R. (2006). Physical Education and Sport in Schools: A Review of Benefits and Outcomes. J. Sch. Health.

[19] Watson A., Timperio A., Brown H., Hesketh K.D. (2017). A Primary School Active Break Programme (ACTI-BREAK): Study Protocol for a Pilot Cluster Randomised Controlled Trial. Trials.

[20] Lynch J., O’Donoghue G., Peiris C.L. (2022). Classroom Movement Breaks and Physically Active Learning Are Feasible, Reduce Sedentary Behaviour and Fatigue, and May Increase Focus in University Students: A Systematic Review and Meta-Analysis. Int. J. Environ. Res. Public Health.

[21] Fiorilli G., Di Claudio G., Di Fonza D., Baralla F., Aquino G., Di Martino G., Della Valle C., Centorbi M., Calcagno G., Buonsenso A. (2025). Active Breaks Enhance Complex Processing Speed, Math Performance, and Physical Activity in Primary School Children: A Randomized Controlled Trial. J. Funct. Morphol. Kinesiol..

[22] Broad A.A., Bornath D.P.D., Grisebach D., McCarthy S.F., Bryden P.J., Robertson-Wilson J., Hazell T.J. (2023). Classroom Activity Breaks Improve On-Task Behavior and Physical Activity Levels Regardless of Time of Day. Res. Q. Exerc. Sport.

[23] Masini A., Salussolia A., Anastasia A., Grao-Cruces A., Soldà G., Zanutto G., Riegger S., Mulato R., Sánchez-Oliva D., Ceciliani A. (2024). Evaluation of School-Based Interventions Including Homework to Promote Healthy Lifestyles: A Systematic Review with Meta-Analysis. J. Public Health.

[24] Latino F., Tafuri F., Maisuradze M., Tafuri M.G. (2025). Enhancing Academic Performance, Cognitive Functions, and Mental Well-Being Through Active Breaks: Evidence from a Pilot Study with University Student Sample. Int. J. Environ. Res. Public Health.

[25] Reyes-Amigo T., Grao-Cruces A., Sanchez-Oliva D., Garcia-Hermoso A., Reyes-Molina D., Yañez-Sepúlveda R., Olivares-Arancibia J., Hurtado-Almonácid J., Páez-Herrera J., Salinas-Gallardo G. (2025). Acute Effect of School-Based Active Breaks on Physical Activity Level and on-Task Classroom Behavior in Primary Schoolchildren. Front. Public Health.

[26] Melguizo-Ibáñez E., Zurita-Ortega F., González-Valero G., Puertas-Molero P., Tadeu P., Ubago-Jiménez J.L., Alonso-Vargas J.M. (2024). Active Break as a Tool for Improving Attention in the Educational Context. A Systematic Review and Meta-Analysis. Rev. Psicodidáctica (Engl. Ed.).

[27] Infantes-Paniagua Á., Silva A.F., Ramirez-Campillo R., Sarmento H., González-Fernández F.T., González-Víllora S., Clemente F.M. (2021). Active School Breaks and Students’ Attention: A Systematic Review with Meta-Analysis. Brain Sci..

[28] Caspersen C.J., Powell K.E., Christenson G.M. (1985). Physical Activity, Exercise, and Physical Fitness: Definitions and Distinctions for Health-Related Research. Public Health Rep..

[29] Lamb K.L., Brodie D.A., Roberts K. (1988). Physical Fitness and Health-Related Fitness as Indicators of a Positive Health State. Health Promot. Int..

[30] Bassett D.R., Fitzhugh E.C., Heath G.W., Erwin P.C., Frederick G.M., Wolff D.L., Welch W.A., Stout A.B. (2013). Estimated Energy Expenditures for School-Based Policies and Active Living. Am. J. Prev. Med..

[31] Reis L.N., Reuter C.P., Burns R.D., Martins C.M.d.L., Mota J., Gaya A.C.A., Silveira J.F.d.C., Gaya A.R. (2024). Effects of a Physical Education Intervention on Children’s Physical Activity and Fitness: The PROFIT Pilot Study. BMC Pediatr..

[32] Wang J., Wu S., Chen X., Xu B., Wang J., Yang Y., Ruan W., Gao P., Li X., Xie T. (2024). Impact of Awareness of Sports Policies, School, Family, and Community Environmental on Physical Activity and Fitness among Children and Adolescents: A Structural Equation Modeling Study. BMC Public Health.

[33] Marrero-Rivera J.P., Sobkowiak O., Jenkins A.S., Bagnato S.J., Kline C.E., Gordon B.D., Taverno Ross S.E. (2024). The Relationship between Physical Activity, Physical Fitness, Cognition, and Academic Outcomes in School-Aged Latino Children: A Scoping Review. Children.

[34] Larose D., Massie C.-L., St-Aubin A., Boulay-Pelletier V., Boulanger E., Lavoie M.D., Yessis J., Tremblay A., Drapeau V. (2024). Effects of Flexible Learning Spaces, Active Breaks, and Active Lessons on Sedentary Behaviors, Physical Activity, Learning, and Musculoskeletal Health in School-Aged Children: A Scoping Review. J. Act. Sedentary Sleep Behav..

[35] Sortwell A., O’Brien K., Murphy A., Ramirez-Campillo R., Piggott B., Hine G., Newton M. (2024). Effects of Plyometric-Based Structured Game Active Breaks on Fundamental Movement Skills, Muscular Fitness, Self-Perception, and Actual Behaviour in Primary School Students. Biol. Sport.

[36] Mendoza-Muñoz M., Calle-Guisado V., Pastor-Cisneros R., Barrios-Fernandez S., Rojo-Ramos J., Vega-Muñoz A., Contreras-Barraza N., Carlos-Vivas J. (2022). Effects of Active Breaks on Physical Literacy: A Cross-Sectional Pilot Study in a Region of Spain. Int. J. Environ. Res. Public Health.

[37] Reyes-Amigo T., Salinas-Gallardo G., Mendoza E., Ovalle-Fernández C., Ibarra-Mora J., Gómez-Álvarez N., Carrasco-Beltrán H., Páez-Herrera J., Hurtado-Almonácid J., Yañez-Sepúlveda R. (2025). Effectiveness of School-Based Active Breaks on Classroom Behavior, Executive Functions and Physical Fitness in Children and Adolescent: A Systematic Review. Front. Public Health.

[38] Porter A., Walker R., House D., Salway R., Dawson S., Ijaz S., de Vocht F., Jago R. (2024). Physical Activity Interventions in European Primary Schools: A Scoping Review to Create a Framework for the Design of Tailored Interventions in European Countries. Front. Public Health.

[39] Whitehead M. (2013). Stages in Physical Literacy Journey. ICSSPE Bull. Sport Sci. Phys. Educ..

[40] Edwards L.C., Bryant A.S., Keegan R.J., Morgan K., Jones A.M. (2017). Definitions, Foundations and Associations of Physical Literacy: A Systematic Review. Sports Med..

[41] Caldwell H.A.T., Di Cristofaro N.A., Cairney J., Bray S.R., MacDonald M.J., Timmons B.W. (2020). Physical Literacy, Physical Activity, and Health Indicators in School-Age Children. Int. J. Environ. Res. Public Health.

[42] Chatoupis C.C. (2018). Physical Education Teachers’ Use of Mosston and Ashworth’s Teaching Styles: A Literature Review. Phys. Educ..

[43] Dudley D.A., Cotton W.G., Peralta L.R. (2015). Teaching Approaches and Strategies That Promote Healthy Eating in Primary School Children: A Systematic Review and Meta-Analysis. Int. J. Behav. Nutr. Phys. Act..

[44] Monacis D., Annoscia S., Limone P., Colella D. (2023). Examining the Effects of Reproductive and Productive Teaching Styles Interventions on Primary Schoolchildren. What Implications for Physical Education Teachers?. Phys. Educ. Theory Methodol..

[45] Monacis D., Pascali G., Haisan A.-A., Bibba M., Colella D. (2026). The Spectrum of Teaching Styles in Physical Education: A Feasibility Study on Children’s Physical Fitness and Self-Perception. Educ. Sci..

[46] Masini A., Marini S., Ceciliani A., Barone G., Lanari M., Gori D., Bragonzoni L., Toselli S., Stagni R., Bisi M.C. (2023). The Effects of an Active Breaks Intervention on Physical and Cognitive Performance: Results from the I-MOVE Study. J. Public Health.

[47] González-Fernández F.T., González-Víllora S., Baena-Morales S., Pastor-Vicedo J.C., Clemente F.M., Badicu G., Murawska-Ciałowicz E. (2021). Effect of Physical Exercise Program Based on Active Breaks on Physical Fitness and Vigilance Performance. Biology.

[48] Faul F., Erdfelder E., Buchner A., Lang A.-G. (2009). Statistical Power Analyses Using G* Power 3.1: Tests for Correlation and Regression Analyses. Behav. Res. Methods.

[49] Faul F., Erdfelder E., Lang A.-G., Buchner A. (2007). G* Power 3: A Flexible Statistical Power Analysis Program for the Social, Behavioral, and Biomedical Sciences. Behav. Res. Methods.

[50] Chiu C.-H., Li J.-Y., Huang W.-C. (2026). Effects of an 8-Week Programmed Physical Activity Intervention on Children’s Cognitive, Emotional, and Body Movement Development—A Quasi-Experimental Study of a Preschool in Taiwan. Children.

[51] Dong H., Wang S. (2026). Impact of Physical Activity on Children’s Cognitive Function and Its Educational Applications: A Narrative Literature Review. Front. Psychol..

[52] Mosston M., Ashworth S. (2008). Teaching Physical Education: 1st Online Edition.

[53] Pill S., SueSee B., Davies M. (2023). The Spectrum of Teaching Styles and Models-Based Practice for Physical Education. Eur. Phys. Educ. Rev..

[54] Ministero dell’Istruzione, dell’Università e Ricerca (2012). Indicazioni Nazionali per Il Curricolo Della Scuola Dell’ Infanzia e Del Primo Ciclo d’ Istruzione.

[55] Council of Europe, Committee for the Development of Sport (1993). Eurofit: Handbook for the Eurofit Tests of Physical Fitness.

[56] Ruiz J.R., Castro-Piñero J., España-Romero V., Artero E.G., Ortega F.B., Cuenca M.M., Jimenez-Pavón D., Chillón P., Girela-Rejón M.J., Mora J. (2011). Field-Based Fitness Assessment in Young People: The ALPHA Health-Related Fitness Test Battery for Children and Adolescents. Br. J. Sports Med..

[57] Falk B., Cohen Y., Lustig G., Lander Y., Yaaron M., Ayalon J. (2001). Tracking of Physical Fitness Components in Boys and Girls from the Second to Sixth Grades. Am. J. Hum. Biol..

[58] Ulrich S., Hildenbrand F.F., Treder U., Fischler M., Keusch S., Speich R., Fasnacht M. (2013). Reference Values for the 6-Minute Walk Test in Healthy Children and Adolescents in Switzerland. BMC Pulm. Med..

[59] Ulrich D.A. (2000). Test of Gross Motor Development 2: Examiner’s Manual.

[60] Kowalski K.C., Crocker P.R.E., Faulkner R.A. (1997). Validation of the Physical Activity Questionnaire for Older Children. Pediatr. Exerc. Sci..

[61] Gobbi E., Elliot C., Varnier M., Carraro A. (2016). Psychometric Properties of the Physical Activity Questionnaire for Older Children in Italy: Testing the Validity among a General and Clinical Pediatric Population. PLoS ONE.

[62] Monacis D., Annoscia S., Colella D., Limone P. (2024). Measuring Validity and Reliability of the Italian Version of Physical Activity Questionnaire for Older Children in Overweight and Obese Children. Front. Educ..

[63] Colella D., Morano M., Bortoli L., Robazza C. (2008). A Physical Self-Efficacy Scale for Children. Soc. Behav. Personal. Int. J..

[64] Carraro A., Young M.C., Robazza C. (2008). A Contribution to the Validation of the Physical Activity Enjoyment Scale in an Italian Sample. Soc. Behav. Pers..

[65] Biancardi A., Stoppa E. (1997). Il test delle campanelle modificato (TCM) una proposta per lo studio dell’attenzione in età evolutiva. Psichiatr. Dell’infanzia E Dell’adolescenza.

[66] Cohen J. (2013). Statistical Power Analysis for the Behavioral Sciences.

[67] Gallè F., Pecoraro P., Calella P., Cerullo G., Imoletti M., Mastantuono T., Muscariello E., Ricchiuti R., Sensi S., Sorrentino C. (2020). Classroom Active Breaks to Increase Children’s Physical Activity: A Cross-Sectional Study in the Province of Naples, Italy. Int. J. Environ. Res. Public Health.

[68] van Stryp O., Duncan M.J., Africa E. (2024). The Effect of Active Brain-Breaks on Fundamental Movement Skills and Executive Functioning of Grade One Children in Cape Town, South Africa. Early Child Dev. Care.

[69] Castillo F., Felin Fochesatto C., de Castro Silveira J.F., Reyes Amigo T., Brand C., Martínez R. (2024). Get Moving and Think Better: Impact of an Intervention Involving Physical Activity and Active Breaks on Children’s Cognition. J. Mov. Health.

[70] Melo J.C.N., Tejada J., Silva E.C.M., Ywgne J., Oliveira D.N., Gandarela L., Silva D.R. (2025). Effects of Physically Active Lessons and Active Breaks on Cognitive Performance and Health Indicators in Elementary School Children: A Cluster Randomized Trial. Int. J. Behav. Nutr. Phys. Act..

[71] Belenguer-Troya A., Estevan I., Romero-Martínez J., Ortega-Benavent N., Montalt-García S., Menescardi C. (2025). The Impact of Active Breaks and Active Learning Intervention on Children’s Physical Activity, Motor Competence, and Physical Literacy: The BALA Study. J. Mot. Learn. Dev..

[72] Latino F., Tafuri F., Saraiello E., Tafuri D. (2023). Classroom-Based Physical Activity as a Means to Improve Self-Efficacy and Academic Achievement among Normal-Weight and Overweight Youth. Nutrients.

[73] Men J., Yu Z., An W., Wang P., Hou X., Zhang Y., Wu S., Zhu G., Wang P., Cui C. (2025). Effects of Exercise on Cardiorespiratory Fitness in Children and Adolescents with Overweight and Obesity: A Systematic Review and Meta-Analysis of 72 Randomized Controlled Trials. BMC Public Health.

[74] Méndez-Giménez A., Pallasá Manteca M., Cecchini J. (2022). Effects of Active Breaks on the Primary Students’ Physical Activity. Rev. Int. Med. Y Cienc. Act. Fis. Y Deport..

[75] Jiménez-Parra J.F., Manzano-Sánchez D., Camerino O., Castañer M., Valero-Valenzuela A. (2022). Incentivar La Actividad Física En El Aula Con Descansos Activos: Un Estudio Mixed Methods. Apunt. Educ. Física Y Deport..

[76] Monacis D., D’arando C., Lucatuorto A., Colella D. (2023). Enhancing Health in Primary School: Unveiling the Impact of the “Physical Snack” Project. J. Phys. Educ. Sport.

[77] Robles-Campos A., Reyes-Molina D., Kracht-Suazo K., Cigarroa I., Cárcamo-Oyarzun J., Martinez-Lopez N., Perez-Ruiz M., Grao-Cruces A., Mota J., Ruiz-Ariza A. (2025). Effects of Video-Guided Active Breaks on Motor Competence of Schoolchildren with Special Education Needs. Children.

[78] Masini A., Longo G., Ricci M., Scheier L.M., Sansavini A., Ceciliani A., Dallolio L. (2024). Investigating Facilitators and Barriers for Active Breaks among Secondary School Students: Formative Evaluation of Teachers and Students. Children.

